# Interpretable Multi-Feature Optical Analysis for Stage- and Batch-Aware Quality Assessment of Brain-Organoid Cultures

**DOI:** 10.3390/s26185971

**Published:** 2026-09-21

**Authors:** Shunxing Bao, Jiteng Xiao, Qiushui Wang, Wenjing Liu, Juan Ren, Ting Chen, Yunhe An

**Affiliations:** Institute of Analysis and Testing, Beijing Center for Physical and Chemical Analysis, Beijing Academy of Science and Technology (Beijing Center for Physical and Chemical Analysis), Beijing 100089, China; baoshunxing@bcpca.ac.cn (S.B.); xiaojiteng0803@126.com (J.X.);

**Keywords:** brain organoids, brightfield optical sensing, artificial intelligence, interpretable machine learning, handcrafted feature extraction, quality assessment, stage-aware analysis, batch generalization

## Abstract

Reliable quality assessment of brain organoids is important for reproducible culture and downstream experimentation, yet routine evaluation relies largely on qualitative brightfield inspection. We investigated whether visual QA criteria could be represented by interpretable image descriptors while accounting for developmental stage and culture batch. We analyzed 692 brightfield image–ROI pairs from six culture batches. Binary QA labels were assigned by one expert. We compared 115 handcrafted features with simple morphology and frozen pretrained DINOv2 and ResNet-50 representations using logistic-regression and random-forest classifiers. Leave-one-batch-out (LOBO) testing was the primary exploratory evaluation; repeated image-level cross-validation and maturation-only analyses provided complementary assessments. Handcrafted logistic regression achieved a pooled LOBO ROC AUC of 0.836 (conditional 95% batch bootstrap interval 0.625–0.960), with substantial variation among held-out batches. Mean repeated-CV AUC was 0.933 across all stages and 0.895 within maturation. At a fixed 0.5 score cutoff, all-stage LOBO sensitivity was 0.656 and specificity was 0.869. The comparison concerns the evaluated frozen-feature pipelines and does not establish superiority over fine-tuned deep learning. These results provide an exploratory image-analysis baseline for brightfield QA; label reproducibility and performance beyond the observed laboratory workflow require further validation.

## 1. Introduction

Brain organoids provide experimentally accessible models of human neurodevelopment and have become increasingly important for studying developmental mechanisms, neurological disorders, and treatment response [1]. Their experimental value, however, depends on reproducible culture quality. Variation can arise across stem-cell lines, differentiation runs, culture batches, developmental stages, and individual organoids, making explicit quality-control procedures necessary for reliable interpretation [2].

Routine quality assessment (QA) commonly begins with visual inspection of brightfield microscopy. Experienced reviewers evaluate characteristics such as successful aggregation, compactness, boundary clarity, internal homogeneity, vacuolation, leakage, and structural integrity. Brightfield imaging is inexpensive, label-free, non-destructive, and readily integrated into daily culture workflows, but visual evaluation remains qualitative and may vary across reviewers. Interpretation is further complicated because normal appearance changes during formation, induction, expansion, and maturation, while culture batches may follow different temporal and morphological patterns [3]. Previous studies nevertheless show that organoid morphology contains biologically and operationally relevant information. Expert-assessed quality has been linked to gross morphology and unintended differentiation, and Feret diameter has been proposed as a practical quality indicator [4]. Hierarchical QC frameworks have likewise placed non-invasive morphology and growth assessment before more detailed cellular or molecular characterization [5]. Protocol-agnostic brightfield screening has further used organoid size, shape, and texture to support morphology-based sample evaluation [6].

Automated brightfield-image analysis has been developed for organoid segmentation, tracking, growth measurement, drug-response assessment, and abnormal-structure recognition. Systems such as OrganoID and OrBITS emphasize individual-organoid detection and longitudinal monitoring [7,8]. Other deep-learning tools classify structural maturity or developmental morphology directly from brightfield images [9,10]. These approaches demonstrate the value of image-based analysis, but they do not fully address routine brain-organoid QA under repeated cross-sectional sampling, where different organoids may be imaged on different culture days and individual trajectories are unavailable. In addition, high-dimensional predictive representations do not directly reveal whether decisions are associated with compactness, boundary irregularity, internal heterogeneity, hollow regions, peripheral leakage, or fragmented components [11]. An interpretable framework that accounts explicitly for developmental stage and culture batch is therefore needed to translate routine visual observations into reproducible quantitative measurements.

Brightfield microscopy records spatial variations in transmitted-light intensity through a microscope and camera-detection chain. In this optical measurement workflow, the acquired image constitutes the measurement output, while computational analysis converts that output into an interpretable QA assessment. The present study develops this analytical component for rapid, label-free, and non-destructive evaluation of organoid structure. Because brightfield imaging is routinely available during culture, it may serve as a structural screening layer that complements higher-content cellular, functional, and molecular measurements [2,12,13,14,15]. The present work does not introduce a new sensor device or evaluate multimodal associations.

**Figure 1:**
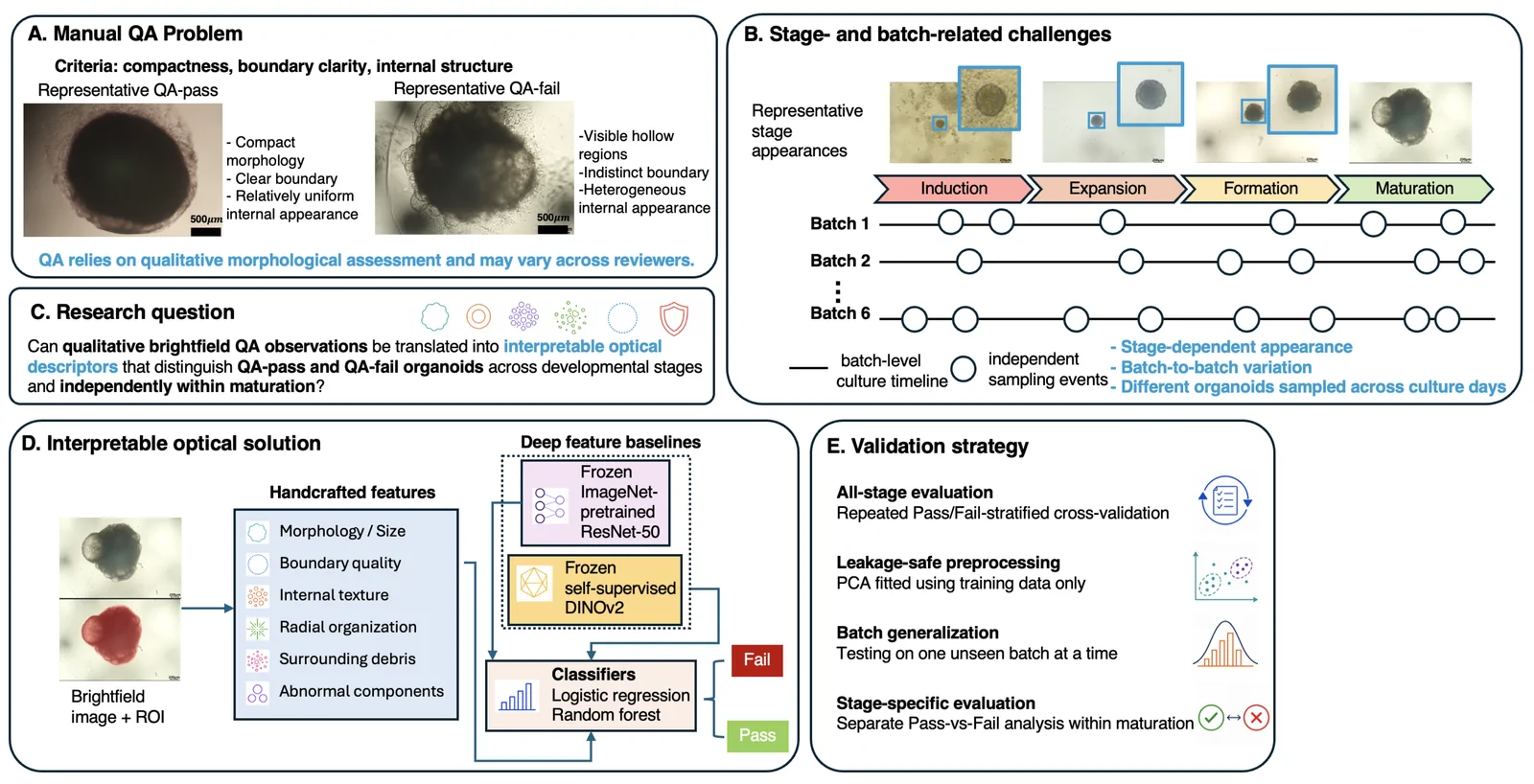
Motivation and study framework for interpretable brightfield organoid quality assessment. (**A**) Routine manual QA is based on qualitative observations of compactness, boundary clarity, and internal appearance. (**B**) Organoid appearance changes across developmental stages and culture batches, and images obtained on different culture days represent independent sampling events rather than longitudinal tracking of the same individual organoid. (**C**) The study asks whether these visual observations can be translated into interpretable optical descriptors across developmental stages and independently within maturation. (**D**) Handcrafted feature families are compared with frozen ImageNet-pretrained ResNet-50 and self-supervised DINOv2 representations using logistic-regression and random-forest classifiers. (**E**) Evaluation includes repeated QA-stratified cross-validation, leakage-safe preprocessing, maturation-only analysis, and leave-one-batch-out testing.

The present study investigates whether qualitative brightfield QA observations can be translated into quantitative and interpretable optical descriptors while explicitly accounting for developmental stage and culture batch. As summarized in Figure 1, brightfield image–ROI pairs from six culture batches were analyzed using domain-informed handcrafted features describing morphology, component-level abnormalities, boundary organization, internal texture, radial organization, and peripheral context. These features were compared with simple morphology and frozen pretrained ResNet-50 [16] and DINOv2 [17] deep representations under a common downstream evaluation framework. The analysis separated the complete all-stage cohort from a maturation-only cohort and used leave-one-batch-out testing as the primary exploratory evaluation, with repeated QA-stratified cross-validation as a secondary assessment of within-dataset discrimination.

## 2. Materials and Methods

### 2.1. Brain-Organoid Culture and Brightfield Image Acquisition

Human pluripotent stem cells (the stem cells used for brain-organoid culture were obtained from the Stem Cell Bank of the Institute of Zoology, Chinese Academy of Sciences, Beijing, China) were maintained in mTeSR complete medium on vitronectin-coated six-well plates at 37 °C in a humidified atmosphere containing 5% CO_2_. Cell handling was performed in a DL-CJ-2NDI clean bench (Harbin Donglian, Harbin, China), and cultures were maintained in a Model 371 CO_2_ incubator (Thermo Scientific, Waltham, MA, USA). During recovery and preparation of a single-cell suspension, Y-27632 was added to the culture medium. To initiate organoid formation, dissociated cells were seeded at 9000 cells per well in round-bottom, ultra-low-attachment 96-well plates and centrifuged at 300× *g* for 5 min using an Allegra X-15R centrifuge (Beckman Coulter, Brea, CA, USA) to promote cellular aggregation.

The culture procedure comprised formation, induction, expansion, and maturation stages. During formation, aggregates were maintained in an AdDMEM/F12-based formation medium containing basic fibroblast growth factor. For neural induction, spheroids were transferred to anti-adherence-treated 24-well plates and cultured in induction medium containing SB431542 and LDN193189, with one or two spheroids placed in each well to reduce contact and fusion. The induced spheroids were embedded in Matrigel and transferred to expansion medium containing basic fibroblast growth factor, epidermal growth factor, and N2 supplement. From maturation onward, organoids were maintained in maturation medium on an orbital shaker at 70 rpm, with medium replacement every 3 days [18].

Brightfield images were collected during routine microscopic inspection using an Observer A1 inverted light microscope (Carl Zeiss, Oberkochen, Germany) with a 4× objective and an E3ISPM20000KPA digital camera. Images were acquired at a resolution of 5440×3648 pixels, corresponding to approximately 1.77 pixels/μm, and recorded in 48-bit RGB format. The camera was operated using automatic exposure and gain control, with a measured exposure time of 28.186 ms, an exposure target value of 120, and gain of 100%. The recorded color temperature was 6503 K with a tint setting of 1000. Focus was adjusted manually. During routine acquisition, focus and illumination intensity were manually adjusted to obtain a clear field of view in which the complete organoid boundary and internal morphology could be visualized. Thus, although camera exposure, gain, and color-balance functions were operated using the software’s automatic modes, illumination intensity and focusing were not fixed across acquisitions.

### 2.2. Study Design and Analytical Cohort

The study included brightfield images from six independent brain-organoid culture batches. Sampling was longitudinal at the batch level but repeated cross-sectional at the individual-organoid level: images acquired on different culture days were not linked by persistent individual-organoid identifiers and were not treated as repeated observations of the same organoid. Each image represented an organoid sampled from a recorded culture day within its corresponding batch. Developmental-stage labels—formation, induction, expansion, and maturation—were taken from the culture records.

**Figure 2:**
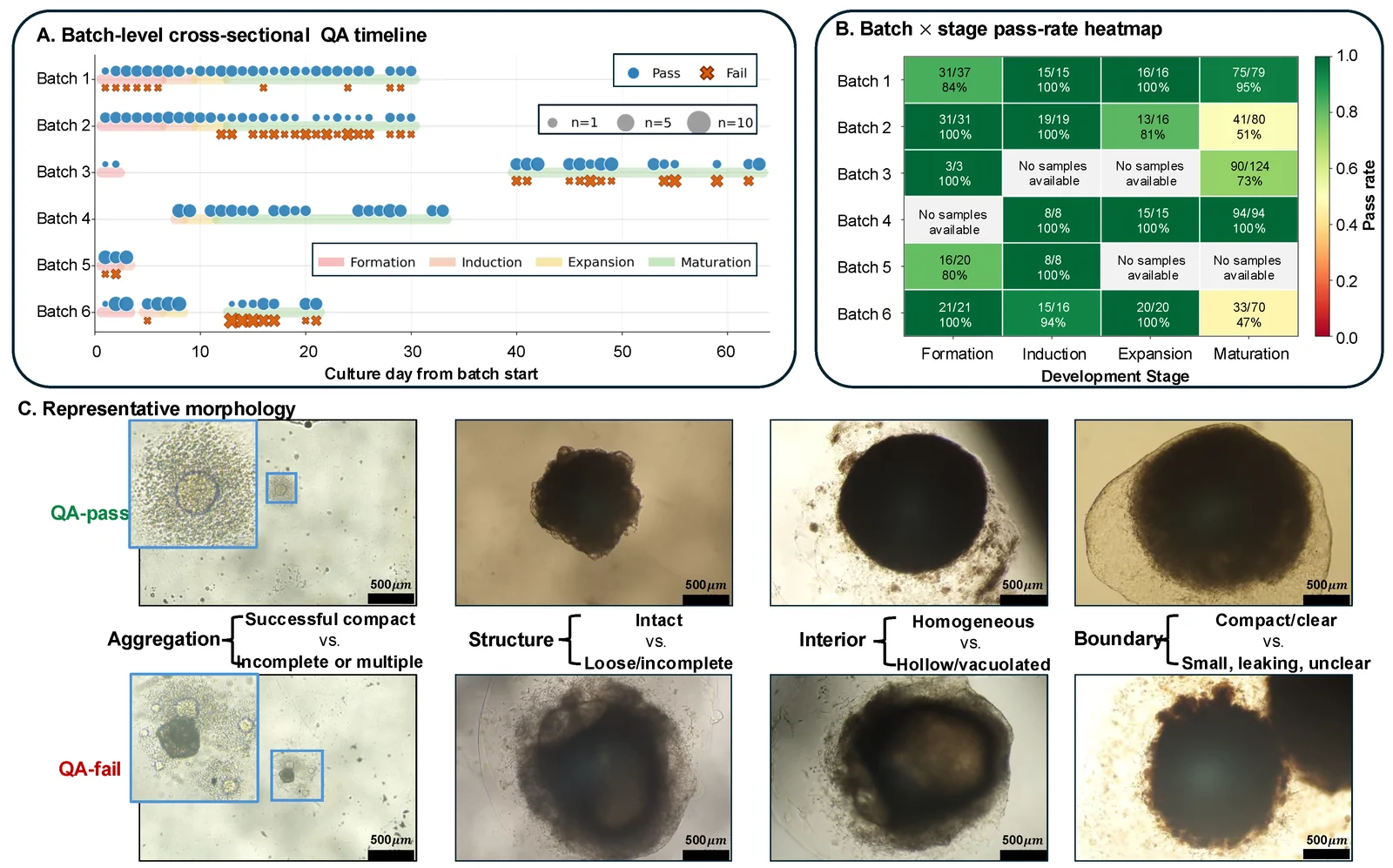
Dataset composition and representative brain-organoid morphology. (**A**) Batch-level repeated cross-sectional QA timeline. Blue circles and orange crosses denote QA-pass and QA-fail observations, respectively; marker size reflects the number of images acquired at a given culture day, and colored timeline segments indicate developmental stage. (**B**) Batch-by-stage pass-rate heatmap showing the number and proportion of QA-pass observations within each available batch–stage combination. (**C**) Representative QA-pass and QA-fail examples illustrating aggregation, structural integrity, internal appearance, and boundary quality.

After matching image records with stage metadata, binary quality-assessment labels, and ROI annotations, the final analytical cohort contained 692 complete image–ROI pairs from six culture batches: 564 QA-pass and 128 QA-fail images. The maturation-only cohort contained 447 images, including 333 QA-pass and 114 QA-fail observations. All image representations were evaluated using these same cohorts. Batch-wise cohort composition is summarized in Table 1; the sampling timeline, stage-specific pass rates, and representative morphology are shown in Figure 2.

Developmental stage and batch identity were used to define analytical subsets, validation strata, and held-out groups; neither variable was included as a predictor in the image-representation models.

### 2.3. Binary Quality-Assessment Labels

Each image had an operational quality label assigned by one expert in the source dataset. The two-expert review described below concerned ROI verification and did not constitute independent QA labeling. Labels were harmonized as QA-pass and QA-fail, with QA-fail treated as the positive class. Visual criteria included cellular aggregation, compactness, structural integrity, boundary clarity, internal homogeneity, hollow or vacuolated regions, peripheral leakage, and adjacent abnormal material. Because expected morphology changed across culture stages, labels were interpreted within the corresponding stage context. Source labels were retained without retrospective relabeling.

### 2.4. QA-Oriented ROI Annotation

Initial ROIs were prepared by a junior annotator who did not know the image-level QA labels, using outputs from in-house organoid segmentation models based on the Segment Anything Model (SAM) [19] and nnU-Net [20] frameworks to assist localization and contour initialization. All ROIs were manually reviewed and adjusted. The completed annotations were verified by two senior domain experts who knew the image-level QA labels.

**Figure 3:**
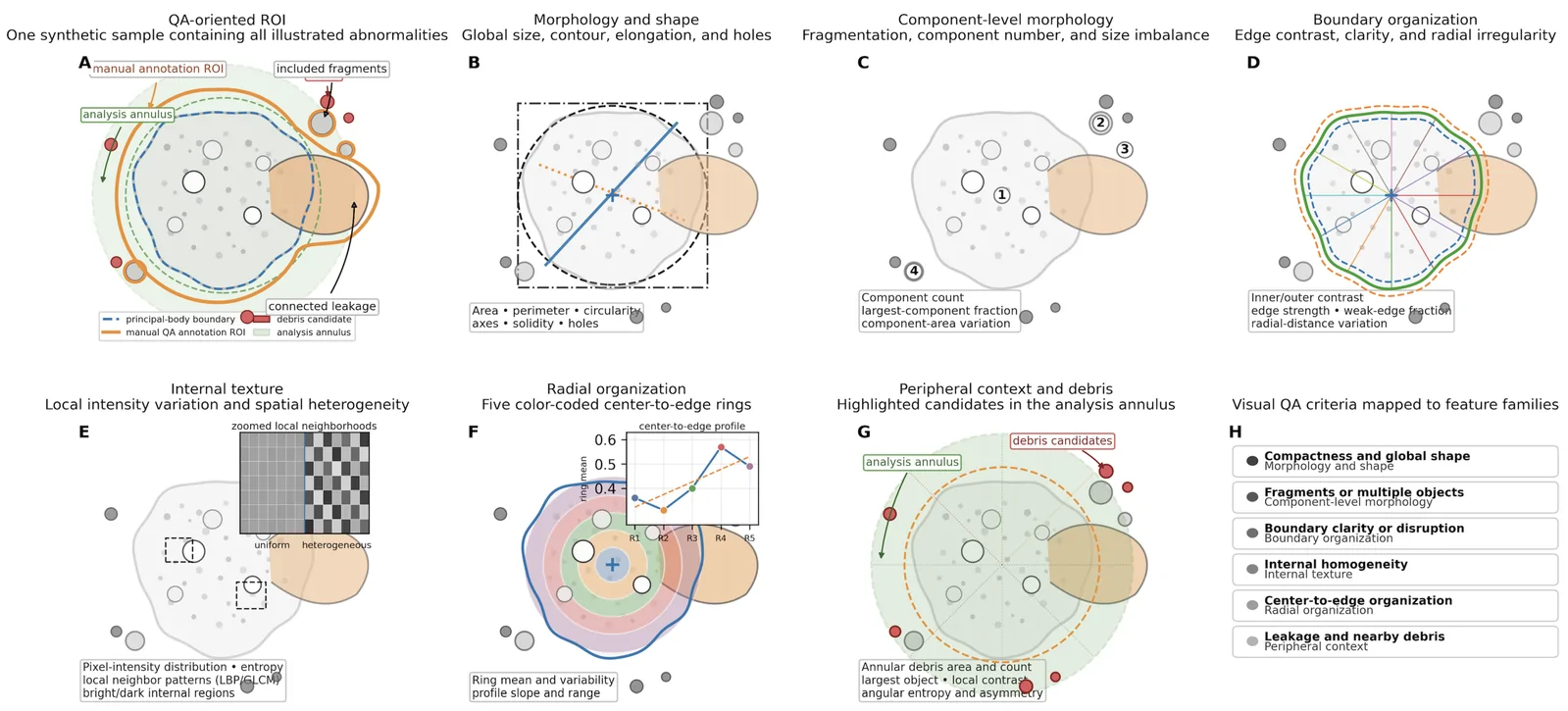
Conceptual illustration of QA-oriented ROI annotation and the six handcrafted feature families. (**A**) The full annotated mask can contain the principal body, connected abnormal extensions and disconnected fragments. (**B**) Morphology and shape are measured on the largest connected component. (**C**) Seven component descriptors use all components in the full mask. (**D**–**F**) Boundary, internal-texture and radial descriptors use the largest component and their specified image regions. (**G**) Peripheral context is measured from the surrounding image annulus and does not require detached material to be separately annotated. (**H**) Mapping between visual QA criteria and feature families. The panels are conceptual illustrations, not evidence that disconnected abnormalities contribute to every feature family.

The annotation target was the QA-relevant image region rather than a strict anatomical boundary, as illustrated in Figure 3A. Each ROI included the principal organoid body and, when present, visually relevant peripheral abnormalities such as leakage-like extensions, protrusions, fragmented components, and nearby debris. An ROI could therefore extend beyond the apparent organoid boundary or contain multiple connected components. Measurements derived from these annotations were treated as optical descriptors of the annotated QA region.

### 2.5. Interpretable Handcrafted Optical Feature Extraction

A total of 115 handcrafted features were extracted from each verified image–ROI pair and organized into six complementary feature families, as summarized in Figure 3B–G: 18 morphology and shape features, 7 component-level morphology and abnormality features, 24 boundary-organization features, 34 internal-texture features, 19 radial-organization features, and 13 peripheral-context features.

Images were converted to grayscale and normalized using the first and 99th intensity percentiles:

(1) $$I_{n}\left(x\right)=\min\left\{1,\max\left\{0,\frac{I\left(x\right)-P_{1}}{P_{99}-P_{1}}\right\}\right\},$$

where $P_{1}$ and $P_{99}$ denote the first and 99th percentiles of finite image intensities. When these percentiles were identical, the observed minimum and maximum were used instead. Per-image percentile normalization standardizes relative intensity range; it is not radiometric calibration or correction for spatially non-uniform illumination. Residual exposure, gain, focus and lighting differences may affect intensity-derived descriptors, which are interpreted as relative appearance measurements.

For intensity-based measurements, a mask-centered crop was generated with padding equal to the larger of 240 native-resolution pixels and 50% of the largest ROI bounding-box dimension. Crops with a maximum side longer than 1600 pixels were downsampled, and binary masks were resized using nearest-neighbor interpolation. Nominal pixel distances used for boundary and peripheral measurements were scaled by the same factor.

#### 2.5.1. Morphology and Shape

Morphology was calculated from the largest connected ROI component at native image resolution. Features included area, perimeter, equivalent diameter, circularity, solidity, eccentricity, major- and minor-axis lengths, aspect ratio, extent, convex area, bounding-box occupancy, convexity-defect measurements, and internal-hole area and count [21].

#### 2.5.2. Component-Level Morphology and Abnormalities

Component-level features were calculated from all connected components in the QA-oriented annotation. If $A_{j}$ denotes the area of component *j*, ordered so that $A_{1}$ is the largest, the largest-component fraction and component-area coefficient of variation were

(2) $$f_{\mathrm{largest}}=\frac{A_{1}}{\sum_{j=1}^{J}A_{j}},\;{\mathrm{CV}}_{A}=\frac{\sigma (A_{1},\ldots ,A_{J})}{\mu (A_{1},\ldots ,A_{J})+\epsilon },$$

where *J* is the number of components and $\epsilon =10^{-8}$. Component count, largest-component area, component-area heterogeneity, and the number of components smaller than 1% of the largest-component area were retained. Seven component descriptors use the full annotated mask. Morphology uses its largest connected component; boundary, texture and radial descriptors use that component and the specified adjacent image regions. Peripheral context is measured from an annulus around the largest component and does not require disconnected regions to be separately annotated.

#### 2.5.3. Boundary Organization

Inner and outer boundary bands were generated by binary erosion and dilation using a nominal native-resolution radius of 6 pixels. Intensity and Sobel-gradient statistics were calculated within the inner, outer, and combined boundary bands, including inner–outer intensity contrast. Boundary irregularity was summarized from centroid-to-contour radial distances using their coefficient of variation and 95th-to-fifth-percentile range. The weak-edge fraction was defined as the proportion of boundary-band pixels with Sobel magnitude no greater than the 25th percentile of finite gradient values in the processed image.

#### 2.5.4. Internal Texture

Within-ROI intensity was summarized by its mean, standard deviation, median, 10th and 90th percentiles, interquartile range, coefficient of variation, and Shannon entropy. Uniform local binary patterns used eight neighbors at radius 1 and were summarized as a normalized 10-bin histogram. Gray-level co-occurrence matrices were calculated after quantization to 32 levels at pixel distances of 1 and 3 and angular offsets of 0, π/4, π/2, and 3π/4. Contrast, dissimilarity, homogeneity, energy, correlation, and angular second moment were summarized across distance–angle combinations [22,23].

Bright and dark internal regions were defined relative to the within-ROI median $m_{\Omega }$ and standard deviation $s_{\Omega }$, using thresholds of $m_{\Omega }+1.5s_{\Omega }$ and $m_{\Omega }-1.5s_{\Omega }$, respectively. Connected regions smaller than 20 processed-image pixels were removed before calculating area fractions and object counts.

#### 2.5.5. Radial Organization

Radial organization was measured relative to the centroid c of the largest ROI component. For pixel x∈Ω, the normalized radial coordinate was

(3) $$\rho \left(x\right)=\frac{{∥x-c∥}_{2}}{\max_{u\in \Omega }{∥u-c∥}_{2}+\epsilon }.$$

The ROI was divided into five equal radial intervals. Mean intensity, intensity standard deviation, and area fraction were calculated in each interval. The retained profile descriptors included the center-to-edge intensity slope, the outermost-minus-innermost intensity difference, and the standard deviation and range of the five ring means.

#### 2.5.6. Peripheral Context and Debris

Peripheral context was evaluated in an annulus extending nominally from 10 to 80 pixels outside the largest ROI component. The background reference region was defined beyond the 80-pixel dilation. Candidate debris pixels were those whose absolute difference from the median background intensity exceeded the 97.5th percentile of background differences. Connected candidate regions smaller than 12 processed-image pixels were removed. Retained measurements included debris area normalized by annular and ROI area, object count, largest-object area, component-area variation, and local contrast. Debris distribution was additionally summarized across eight angular sectors using angular entropy and the largest-sector fraction.

### 2.6. Frozen Deep-Feature Representations

Two pretrained encoders were included as fixed comparative representations: a self-supervised DINOv2 vision transformer and an ImageNet-supervised [24] ResNet-50 convolutional neural network. Because the cohort contained 694 images, encoder weights were not fine-tuned; only fixed feature vectors were extracted.

For each image, the bounding box of the largest ROI component was expanded by 25% of its longest side. The unmasked crop was resized to 224×224 pixels, converted to three channels when necessary, and normalized using the ImageNet channel means and standard deviations.

DINOv2 features were extracted using a frozen ViT-S/14 encoder, retaining the normalized final class token as a 384-dimensional vector. ResNet-50 features were extracted using ImageNet-1K V2 weights, replacing the classification layer with an identity operation and retaining the 2048-dimensional global-average-pooled vector. All encoder parameters remained fixed.

### 2.7. Classification Models and Leakage-Safe Preprocessing

Five input representations were evaluated:

- 12-variable simple-morphology baseline;
- 115-feature handcrafted representation;
- 384-dimensional DINOv2 features;
- 2048-dimensional ResNet-50 features;
- A combined handcrafted–DINOv2 representation.

The simple-morphology baseline contained area, perimeter, equivalent diameter, circularity, solidity, eccentricity, major- and minor-axis lengths, aspect ratio, extent, hole-area fraction, and hole count. The same observations and data partitions were used for all representations.

All data-dependent preprocessing was fitted within each training fold. Nonfinite values were treated as missing and replaced using training-fold medians. Features were standardized using training-fold means and standard deviations, and the fitted transformations were then applied to the corresponding held-out data.

Logistic regression was the primary classifier. It used $L_{2}$ regularization, balanced class weights, the liblinear solver, C=1.0, and a maximum of 5000 iterations; the dual formulation was enabled when the transformed feature dimension exceeded the number of training observations. Predicted QA-fail probability was used as the continuous model score. Individual $L_{2}$ coefficients are conditional on the correlated representation and were not interpreted as independent feature contributions.

A random forest was evaluated as a nonlinear sensitivity model using 500 trees, bootstrap sampling, balanced-subsample class weights, square-root feature selection at each split, a minimum of two samples per terminal leaf, and no maximum tree depth [25].

Principal component analysis (PCA) was evaluated for the handcrafted, DINOv2, and ResNet-50 representations. PCA was fitted only to standardized training data, using randomized singular-value decomposition and

(4) $$K=\min\left(64,\;n_{\mathrm{train}}-1,\;p\right),$$

where $n_{\mathrm{train}}$ is the number of training observations and *p* is the input dimension. The fitted transformation was applied to held-out data [21].

For the combined handcrafted–DINOv2 representation, the handcrafted block was median-imputed and standardized without PCA. The DINOv2 block was independently imputed, standardized, and reduced to at most 64 principal components within the training fold. The transformed blocks were then concatenated before classifier fitting.

As a sensitivity analysis, constant columns and redundant features were removed within each training split using a greedy absolute Spearman-correlation threshold of 0.90. Feature selection, imputation, scaling and model fitting used training data only. Correlation pruning assessed sensitivity to redundant predictors and did not identify independent biological effects.

### 2.8. Validation Design and Statistical Analysis

Model performance was evaluated separately in the all-stage cohort (n=692) and maturation-only cohort (n=447). Complete-batch holding out was the primary exploratory evaluation. Repeated image-level cross-validation provided a secondary estimate of within-dataset performance.

#### 2.8.1. Repeated Stratified Cross-Validation

Within-cohort performance was evaluated using QA-stratified five-fold cross-validation repeated with five random seeds (7, 17, 27, 37, and 47). Joint QA-by-stage stratification was not feasible because at least one joint stratum contained fewer than two observations; the implemented all-stage splitter therefore used QA-class stratification. The maturation-only cohort was likewise stratified by QA class. Developmental stage was addressed through the separate all-stage and maturation-only analyses and was not supplied to the classifier.

For each seed, the five held-out folds were concatenated to obtain one complete out-of-fold prediction set. Mean seed-specific ROC AUC, standard deviation and range were used to describe partition sensitivity. The five seeds reused the same observations and were not treated as independent experiments. AUC calculated after averaging the five held-out scores per image was labeled separately from mean seed-specific AUC.

#### 2.8.2. Leave-One-Batch-Out Evaluation

Performance on held-out culture batches was assessed using leave-one-batch-out (LOBO) cross-validation. In each fold, one batch was held out while the classifier and all data-dependent preprocessing were fitted on the remaining batches.

Per-batch ROC AUC was reported only when both QA classes were present. Single-class batches remained in pooled predictions and threshold-based evaluations. A batch without maturation observations was excluded from the maturation-only analysis. Conditional 95% intervals used 2000 class-stratified image-bootstrap samples for per-batch AUCs and 2000 complete-batch bootstrap samples for pooled LOBO AUCs. Fitted predictions were held fixed; these intervals do not include uncertainty from retraining models on newly sampled datasets.

#### 2.8.3. Performance Measure

QA-fail was defined as the positive class. Model discrimination was summarized using ROC AUC and non-interpolated average precision. At a fixed score cutoff of 0.5, we additionally report sensitivity, specificity, positive predictive value (PPV), negative predictive value (NPV), balanced accuracy, F1 score, false-negative rate, false-positive rate, and confusion-matrix counts. The false-negative rate was calculated as FN/(TP+FN), and the false-positive rate as FP/(TN+FP). The 0.5 cutoff was used as a descriptive reference point and was not optimized as a deployment threshold. Selection of an operational threshold would require tuning using training data according to the relative costs of missed failures and false alarms, followed by independent evaluation.

#### 2.8.4. Statistical Analysis

Paired AUC differences were calculated from aligned held-out predictions for the same images. Conditional 95% percentile intervals used 2000 complete-batch bootstrap samples, with the same sampled batches for both models. Fitted predictions were held fixed; these intervals do not include full model-retraining uncertainty. Repeated seeds were not treated as independent experiments, and paired seed-level or batch-level t-tests were not used to support superiority claims.

Feature distributions were displayed by developmental stage and QA label as descriptive analyses. Coefficient-ranking claims and the previous stratified feature-comparison tests were not retained in the revised presentation. These distributions do not identify independent biological effects or remove confounding by culture age, batch or acquisition conditions.

All analyses were implemented in Python 3.10.20 using NumPy 2.2.6, pandas 2.3.3, scikit-learn 1.7.2, PyTorch 2.5.1, and torchvision 0.20.1. Frozen deep-feature extraction was performed using an NVIDIA GeForce RTX 4070 Ti GPU (NVIDIA Corporation, Santa Clara, CA, USA).

#### 2.8.5. Stage and Representation Sensitivity Analyses

Feature distributions were displayed by stage and QA label, and separate formation and maturation classifiers were evaluated. Induction and expansion were not fitted separately because they contained only one and three QA-fail images. Formation results were interpreted cautiously because its ten failures occurred in only two batches. To assess the contribution of disconnected annotations, models were refitted after retaining only the largest connected component of each mask. The original image context and remaining feature-extraction settings were retained. This counterfactual changes component descriptors without testing alternative boundaries of the principal body. Training-only correlation pruning was evaluated separately as described in Section 2.7.

### 2.9. Use of Generative Artificial Intelligence

Generative AI tools were used only as coding and manuscript-preparation aids. OpenAI ChatGPT and Google Gemini assisted with drafting and refining selected Python and Bash scripts used in the computational workflow. Each AI-assisted script was manually reviewed, executed, tested, and revised by the authors before inclusion in the final analysis pipeline. The authors retained full control over method selection, analytical decisions, output verification, and interpretation. Generative AI was not used to generate or modify experimental data, microscopy images, annotations, class labels, or quantitative results. OpenAI ChatGPT (OpenAI, San Francisco, CA, USA; https://chatgpt.com, accessed on 15 September 2026) was used during July–September 2026 to generate non-data-bearing conceptual icons for Figure 1C–E.

## 3. Results

### 3.1. Distribution of QA Outcomes Across Developmental Stages and Culture Batches

Among the 128 QA-fail observations in the complete analytical cohort, 114 (89.1%) occurred during maturation (Figure 2). The culture batches differed in observation period and sampling density. Batch 3 contained only formation-stage observations, whereas the remaining batches covered different combinations of formation, induction, expansion, and maturation.

Maturation pass rates were 95% in Batch 1 (75/79), 73% in Batch 2 (90/124), 100% in Batch 4 (94/94), 47% in Batch 5 (33/70), and 51% in Batch 6 (41/80). Batch 3 contained no maturation observations. Representative QA-pass and QA-fail images are shown in Figure 2C.

### 3.2. Interpretable Optical Features Associated with QA Status

**Figure 4:**
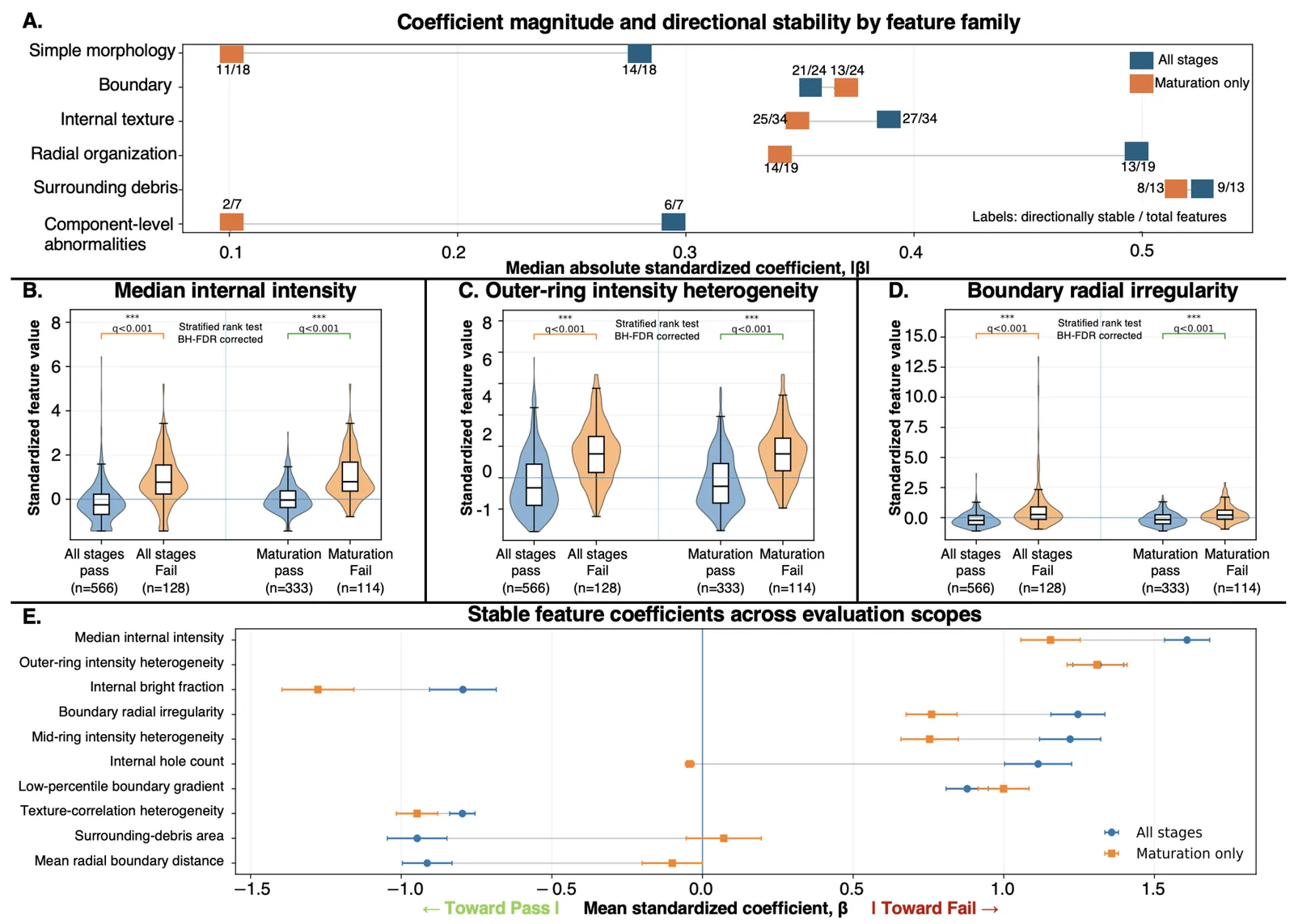
Interpretable optical features associated with organoid QA. (**A**) Median absolute standardized logistic-regression coefficient by feature family for the all-stage and maturation-only analyses. Labels report the number of directionally stable features relative to the total number of features in each family. (**B**–**D**) Distributions of median internal intensity, outer-ring intensity heterogeneity, and boundary radial irregularity for QA-pass and QA-fail images. Displayed *q*-values were obtained from stratified rank tests with Benjamini–Hochberg false-discovery-rate correction; *** denotes q < 0.001. (**E**) Mean standardized coefficients and 95% intervals for selected features across the two analytical scopes. Positive coefficients indicate association with QA-fail, and negative coefficients indicate association with QA-pass.

Coefficient magnitude and directional stability differed across feature families (Figure 4A). In the all-stage analysis, 14 of 18 simple-morphology features, 21 of 24 boundary features, 27 of 34 internal-texture features, 13 of 19 radial-organization features, 9 of 13 surrounding-debris features, and 6 of 7 component-level abnormality features retained the same coefficient direction in at least 80% of the 25 fitted models. Within maturation, the corresponding counts were 13/18, 13/24, 25/34, 14/19, 8/13, and 2/7. The largest median absolute standardized coefficients occurred in the radial-organization and surrounding-debris families in the all-stage analysis.

Three representative features were higher in QA-fail images after stratified comparison. Median internal intensity showed stratified rank-biserial effects of 0.528 across all stages and 0.561 within maturation. Outer-ring intensity heterogeneity showed corresponding effects of 0.423 and 0.432, while boundary radial irregularity showed effects of 0.281 and 0.267. All six comparisons remained significant after Benjamini–Hochberg correction (all q<0.001; Figure 4B–D).

Selected standardized coefficients are shown in Figure 4E. Higher median internal intensity, outer-ring intensity heterogeneity, boundary radial irregularity, mid-ring intensity heterogeneity, and low-percentile boundary gradient were associated with QA-fail in both analytical scopes. Internal bright fraction and texture-correlation heterogeneity were associated with QA-pass in both scopes. Surrounding-debris area showed a directionally stable negative coefficient in the all-stage analysis but was not directionally stable within maturation.

### 3.3. Comparison of Handcrafted and Frozen Deep Representations

Across all stages, mean repeated-CV ROC AUCs for logistic regression were 0.761 for simple morphology, 0.933 for handcrafted features, 0.892 for frozen DINOv2, and 0.833 for frozen ResNet-50. Combining handcrafted and DINOv2 features gave an AUC of 0.932. Within maturation, the corresponding AUCs were 0.692, 0.895, 0.868, 0.815, and 0.896. The observed differences describe these fixed pipelines and do not establish a general ranking against task-adapted deep networks.

**Figure 5:**
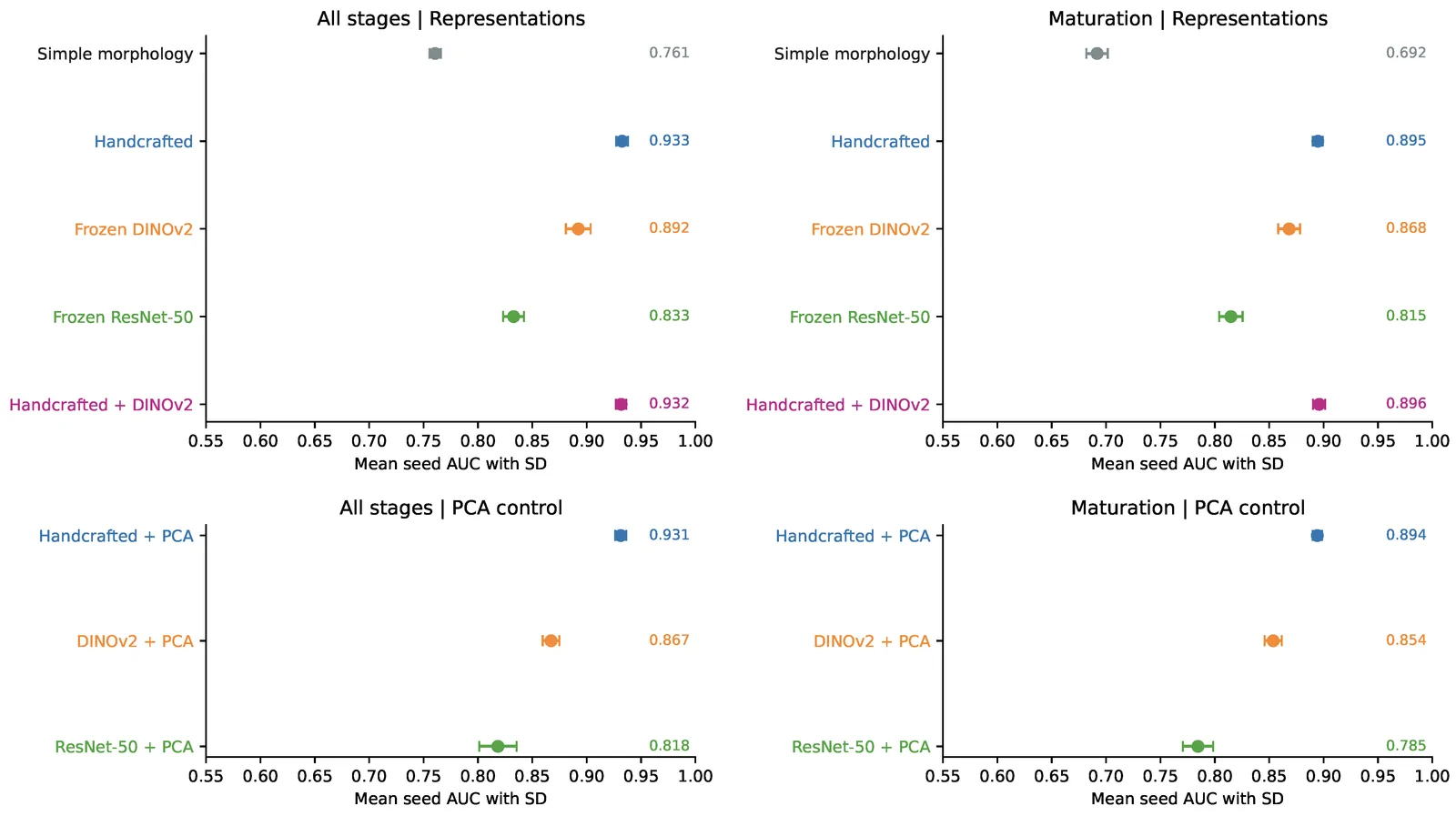
Comparison of image representations under repeated stratified five-fold cross-validation across five seeds using logistic regression. The upper-left and upper-right panels show all-stage and maturation-only results; the lower panels show the corresponding training-fold PCA controls. Points indicate mean seed-specific ROC AUC and horizontal bars indicate standard deviations. The encoders were frozen, and seed variation is not treated as independent experimental replication.

With training-fold PCA to 64 components, all-stage AUCs were 0.931 for handcrafted features, 0.867 for DINOv2, and 0.818 for ResNet-50. Maturation-only AUCs were 0.894, 0.854, and 0.785, respectively. Figure 5 reports mean seed-specific AUCs with standard deviations. Table 2 provides the LR/RF comparison, including pooled LOBO results.

Under random-forest evaluation, mean repeated-CV ROC AUC was 0.926 for handcrafted features, 0.885 for DINOv2 and 0.856 for ResNet-50 across all stages. Within maturation, the corresponding values were 0.888, 0.868 and 0.822 (Table 2).

### 3.4. Performance on Held-Out Culture Batches

**Figure 6:**
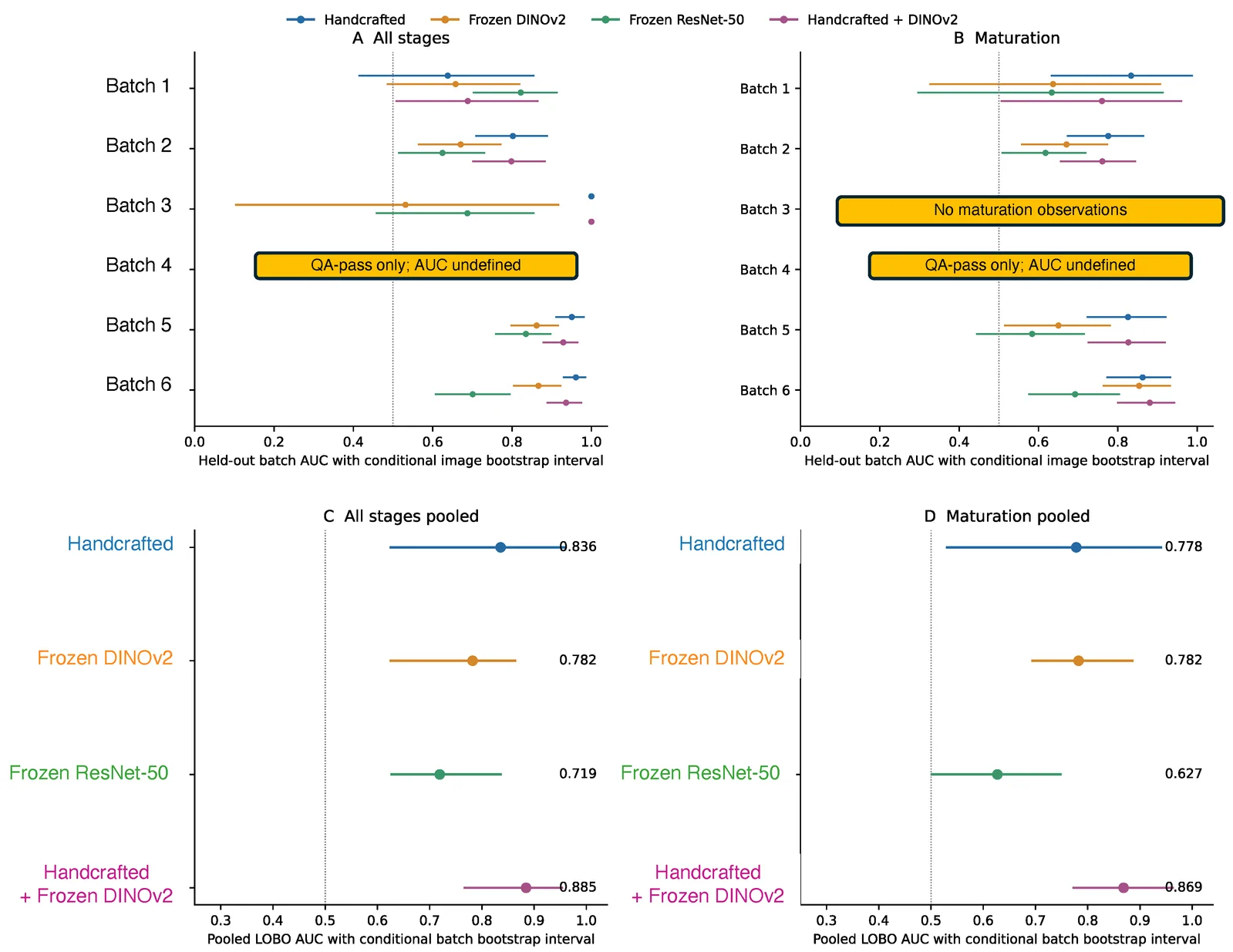
Performance on held-out culture batches using logistic regression. (**A**,**B**) Per-batch ROC AUCs for the all-stage and maturation-only analyses, with conditional 95% intervals obtained from 2000 class-stratified bootstrap resamples of images within each held-out batch. Batch 4 contained only QA-pass observations and therefore had no estimable within-batch AUC; Batch 3 contained no maturation observations. (**C**,**D**) Pooled LOBO ROC AUCs with conditional 95% intervals obtained from 2000 complete-batch bootstrap resamples. Fitted predictions were held fixed during resampling; therefore, the intervals do not include model-retraining uncertainty. The dashed vertical line indicates an AUC of 0.5.

The primary handcrafted logistic-regression pooled LOBO AUC was 0.836 (conditional 95% interval 0.625–0.960) across all stages and 0.778 (0.531–0.940) within maturation (Figure 6C,D). All-stage per-batch AUCs ranged from 0.638 to 1.000 across five estimable batches (Figure 6A). Batch 3 had only four QA-fail images; its perfect ranking and degenerate conditional interval do not establish precise performance. Batch 4 contained only QA-pass observations and therefore had no estimable within-batch AUC. Within maturation, AUC was estimable in four batches and ranged from 0.776 to 0.862 (Figure 6B).

All-stage pooled LOBO AUCs were 0.782 for frozen DINOv2, 0.719 for frozen ResNet-50, and 0.885 for handcrafted plus DINOv2 (Figure 6C). Within maturation, these values were 0.782, 0.627, and 0.869, respectively (Figure 6D). For all-stage logistic regression, paired AUC differences for handcrafted minus DINOv2 and handcrafted minus ResNet-50 were 0.054 (−0.033 to 0.154) and 0.117 (−0.076 to 0.270). These conditional intervals and the variation among held-out batches limit comparative and generalization claims.

### 3.5. Operating-Point Performance

**Figure A1:**
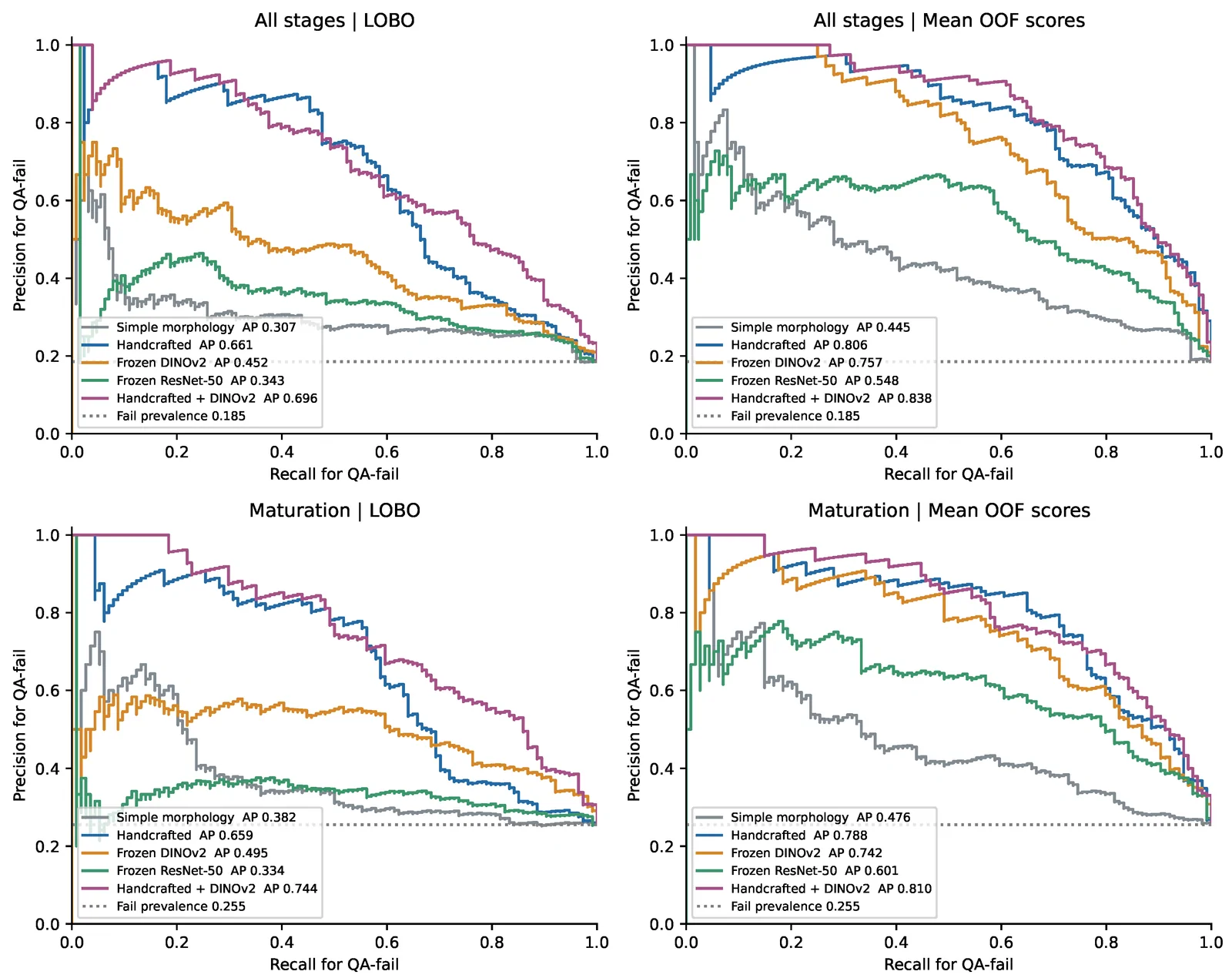
Precision–recall curves for the logistic-regression pipelines, with QA-fail defined as the positive class. Panels show pooled LOBO predictions and averages of repeated-CV held-out scores for the all-stage and maturation-only analyses. The horizontal reference indicates the prevalence of QA-fail. AP denotes non-interpolated average precision.

**Figure A2:**
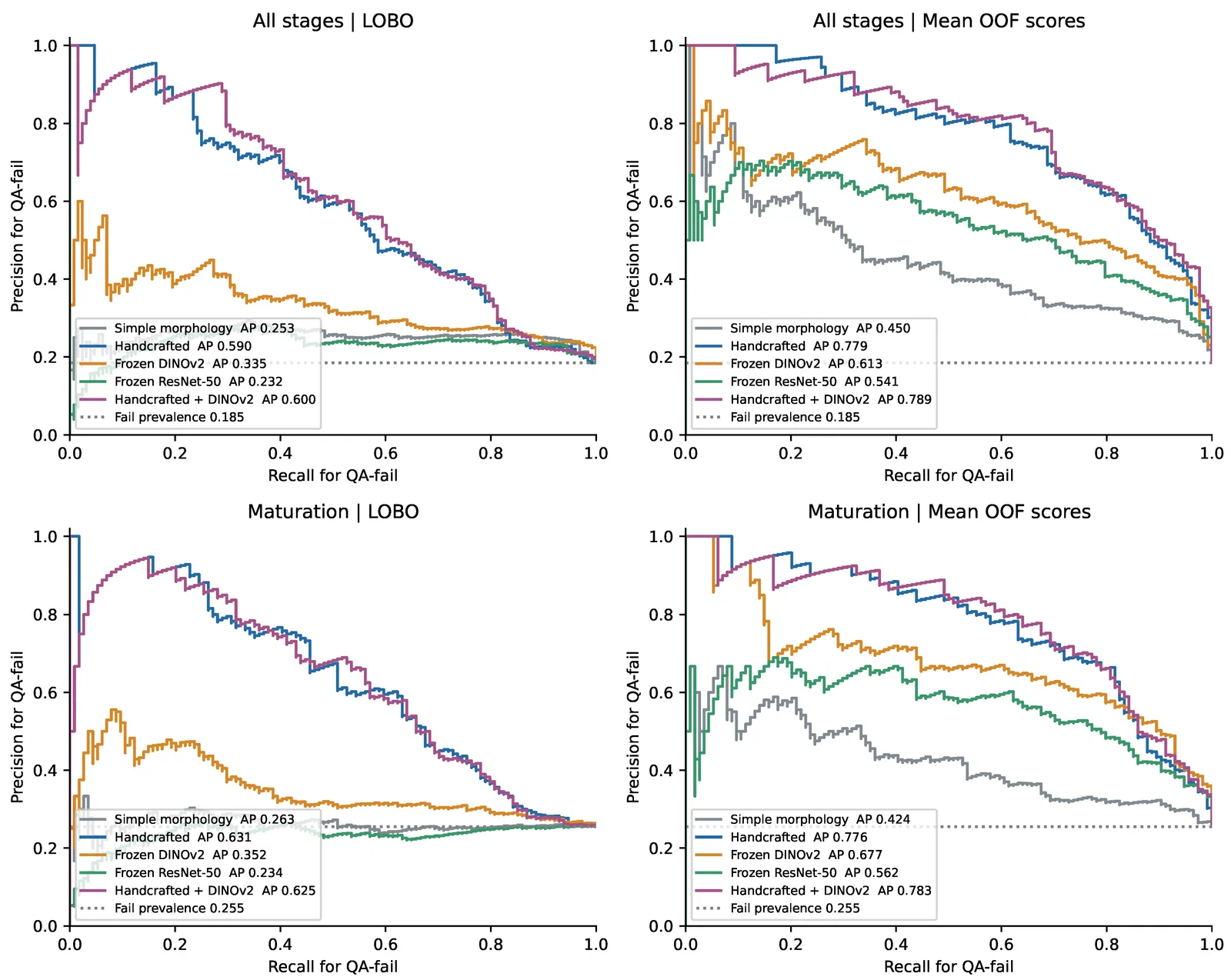
Precision–recall curves for the random-forest pipelines, with QA-fail defined as the positive class.

**Figure A3:**
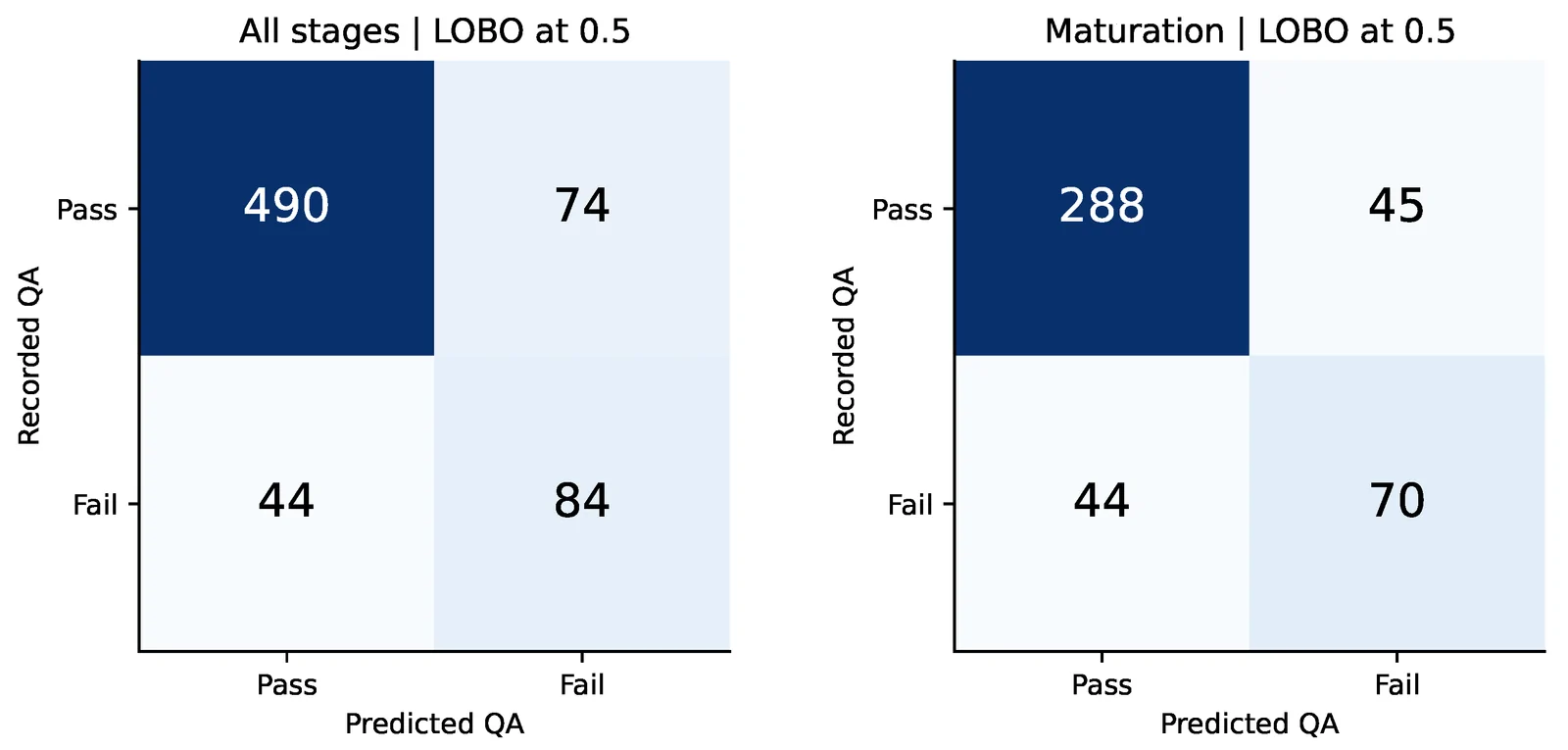
Confusion matrices for the handcrafted logistic-regression model based on pooled LOBO predictions at the fixed score cutoff of 0.5.

At the fixed score cutoff of 0.5, pooled all-stage LOBO predictions from the handcrafted logistic-regression model gave TN = 490, FP = 74, FN = 44, and TP = 84. The model therefore identified 84 of 128 QA-fail observations and 490 of 564 QA-pass observations. Sensitivity was 0.656, specificity 0.869, PPV 0.532, NPV 0.918, balanced accuracy 0.763, and F1 score 0.587. The false-negative and false-positive rates were 0.344 and 0.131, respectively. Within maturation, the corresponding counts were TN = 288, FP = 45, FN = 44, and TP = 70, with a sensitivity of 0.614 and specificity of 0.865. Table 3 provides the complete operating-point results, while Appendix A, Figure A1, Figure A2 and Figure A3 show precision–recall curves and confusion matrices, respectively. Because the reference cutoff was not optimized for deployment, these findings should be interpreted as descriptive operating-point performance. In particular, the observed false-negative rate does not support use of the model as a stand-alone automated QA decision rule without threshold optimization and independent validation.

### 3.6. Stage and ROI Sensitivity Analysis

**Figure A4:**
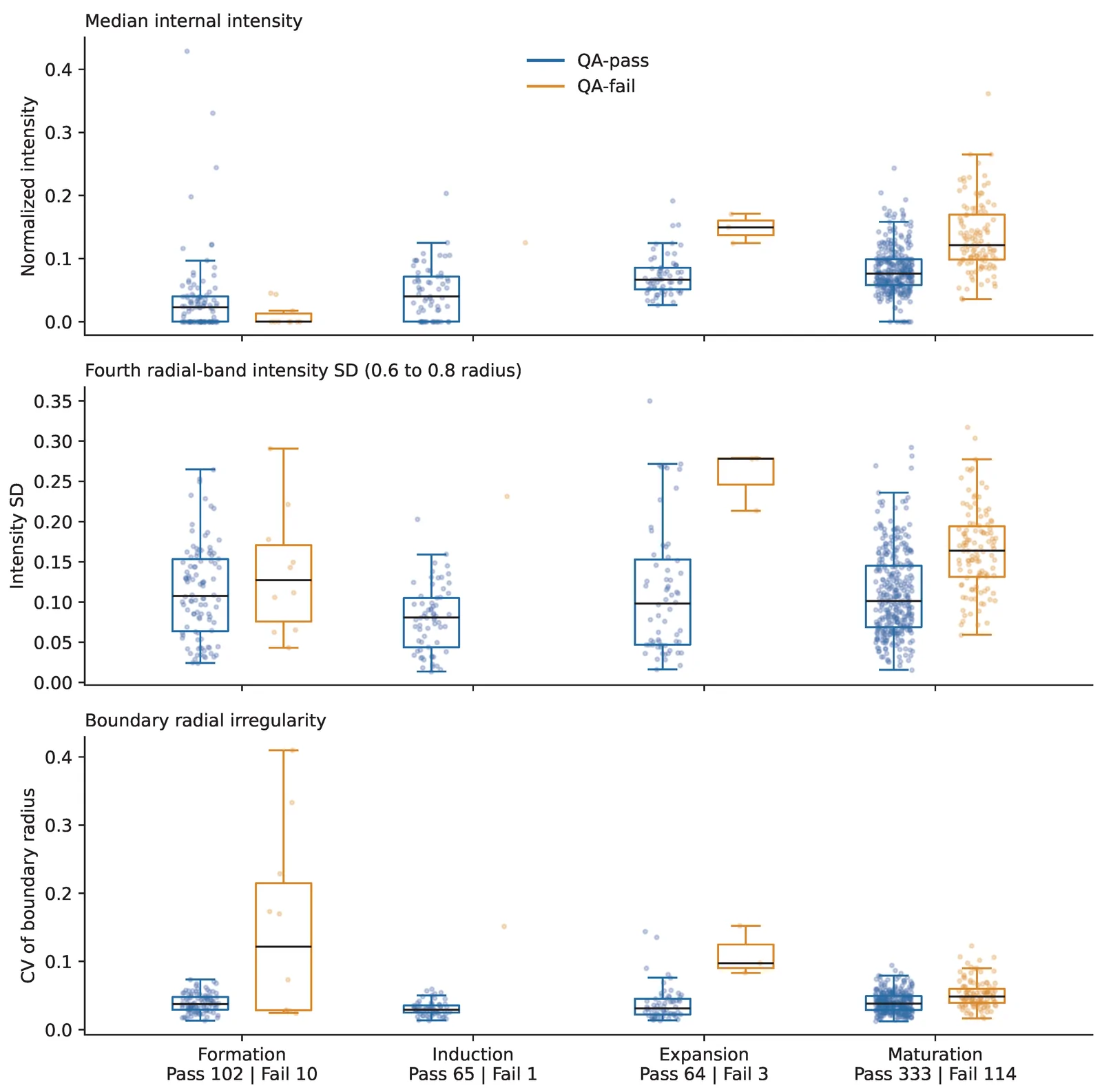
Distributions of selected handcrafted descriptors and QA counts by developmental stage. These descriptive associations do not remove within-stage age, batch or acquisition effects.

Formation included 112 images from five batches, with ten QA-fail images confined to two batches. Formation-only handcrafted LR achieved mean repeated-CV AUC 1.000, but pooled LOBO AUC was 0.754 (conditional interval 0.422–1.000). Induction and expansion contained only one and three QA-fail images and were not fitted separately. Feature distributions by stage and QA status are shown in Figure 4 and Figure A4.

Only six of the 692 masks contained disconnected components, all in formation-stage QA-fail images. A largest-component-only mask counterfactual gave all-stage pooled LOBO AUC 0.800, compared with 0.836 for the full mask. The paired difference was 0.036 (conditional interval 0.002–0.192). Within maturation, features and predictions were identical because every mask had one component. This limited comparison does not establish a general advantage over main-body segmentation or exclude bias from unblinded expert ROI verification.

## 4. Discussion

### 4.1. Principal Findings

This study examined whether routine visual QA observations in brain-organoid brightfield microscopy could be represented using quantitative and interpretable optical descriptors. Three findings were consistent across the completed analyses. First, QA-fail observations were concentrated during maturation, while maturation pass rates differed among culture batches. Second, the complete handcrafted representation achieved higher repeated-cross-validation ROC AUC than simple morphology, DINOv2, and ResNet-50, whereas adding DINOv2 features did not improve performance. Third, the handcrafted representation retained discrimination under leave-one-batch-out (LOBO) evaluation, although performance varied across held-out batches and the corresponding confidence intervals were broad.

These findings support a limited, dataset-specific conclusion. Under the imaging protocol, operational QA labels, and validation procedures used here, domain-informed optical descriptors captured QA-relevant information more effectively and more transparently than the two frozen pretrained representations. This comparison does not establish that handcrafted features are universally superior to deep learning, and it does not address the performance of task-specific fine-tuning, other pretrained encoders, or larger multi-institutional training collections.

### 4.2. Interpretability of Brightfield QA Signals

The feature analysis describes internal intensity, fourth radial-band intensity variability and boundary radial irregularity by developmental stage and QA label. Interpretability refers to the explicit definitions of these image measurements and inspection of their distributions. The fourth radial band spans normalized radii 0.6–0.8 and is not the outermost band.

Correlated predictors can redistribute L2 coefficient magnitudes and signs. Neither directional stability nor correlation pruning establishes an independent biological contribution for an individual descriptor. Brightfield intensity also depends on illumination, focus, optical path length, local thickness and internal organization, preventing a unique cellular or molecular interpretation.

Previous brain-organoid studies have linked gross morphology to expert QA and aspects of tissue development while recognizing its biological limitations [3,4,5]. The stage-specific distributions and separate maturation models provide complementary descriptions of the recorded labels, but do not remove age, batch or acquisition effects within a stage.

### 4.3. Importance of Stage- and Batch-Aware Evaluation

The dataset was longitudinal at the culture-batch level but repeated cross-sectional at the organoid level. Observations collected on different culture days did not represent repeated measurements of the same individual organoid. The timeline in Figure 2 should therefore be interpreted as the changing composition of sampled organoids within each batch rather than as individual growth trajectories. Methods designed for tracked objects would require assumptions that are not supported by this sampling process.

Mean repeated-CV AUC was 0.933 across all stages and 0.895 within maturation; the corresponding pooled LOBO AUCs were 0.836 and 0.778. Maturation-only analysis addresses differences between developmental stages, but does not remove variation in culture age, batch or acquisition conditions within maturation. Because the cohorts overlap and differ in composition, their AUC difference should not be interpreted as a quantitative estimate of the contribution of developmental stage.

LOBO evaluation exposed an additional level of variability. Handcrafted performance was high for several held-out batches but lower for others, and one batch contained only QA-pass observations. The wide batch-clustered confidence intervals reflect the small number of independent batches and their heterogeneous class composition. Pooled LOBO estimates summarize all held-out predictions, whereas per-batch estimates show how performance varies across individual culture runs. This variability is consistent with broader concerns regarding batch effects, reporting, and reproducibility in brain-organoid research [2].

### 4.4. Handcrafted Features and Frozen Deep Representations

The complete handcrafted representation achieved higher mean repeated-cross-validation ROC AUC than either evaluated frozen deep representation in both analytical scopes. The handcrafted features incorporated organoid-specific QA knowledge, whereas the encoders were not adapted to the organoid images or labels. Matching partitions and downstream classifiers does not equalize task-specific prior information or image-processing choices. The comparison therefore concerns the specified pipelines and does not establish superiority over task-specific fine-tuning or other microscopy-pretrained encoders.

Combining handcrafted and DINOv2 features gave mean repeated-CV AUCs of 0.932 across all stages and 0.896 within maturation, compared with 0.933 and 0.895 for handcrafted features alone. The combined representation had numerically higher pooled LOBO AUCs of 0.885 and 0.869. The small number of batches and wide conditional intervals limit conclusions about incremental benefit.

The PCA sensitivity analysis provides a further control for raw feature dimension. Applying fold-specific PCA with a common maximum of 64 components left handcrafted performance essentially unchanged, while DINOv2 and ResNet-50 performance decreased in several settings. The similar handcrafted performance before and after PCA suggests that its relative performance was not attributable only to the number of input variables. This analysis does not identify an optimal feature dimension or determine how other dimensionality-reduction strategies would perform.

### 4.5. Interpretation of QA-Oriented ROIs

The annotations represented QA-relevant image regions rather than strict anatomical boundaries. Their disconnected components contribute through seven component descriptors; most remaining measurements use the largest connected component or its adjacent image regions. Morphology therefore describes the principal annotated component, including any connected abnormal extensions, rather than the sum of all disconnected material.

Only six masks contained disconnected components, all in formation-stage QA-fail images. The largest-component-only counterfactual reduced all-stage pooled LOBO AUC from 0.836 to 0.800, while maturation features and predictions were identical. This supports a limited, stage-dependent contribution and does not establish a general advantage over main-body segmentation. The counterfactual does not test alternative main-body boundaries or remove potential bias from unblinded expert verification.

### 4.6. Relationship to Broader Optical and Multimodal Characterization

Only brightfield images were included in the analyses reported here; other imaging, molecular, spectroscopic, or functional measurements were outside the scope of the current study. Brightfield microscopy provides a non-destructive view of gross structure and optical appearance, whereas complementary measurements can characterize cellular identity, molecular composition, spatial biochemistry, or electrophysiological function. Brain-organoid quality frameworks therefore commonly place morphology within a broader set of structural and biological measurements rather than treating it as a complete description of organoid state [2,3].

The present framework is complementary to comprehensive organoid-image-analysis systems such as BrAIn, which combine morphology classification, segmentation, object detection, and developmental measurements [10]. Rather than developing a multi-task image-analysis application, our analysis focused on translating routine operational QA criteria into named optical descriptors and evaluating their behavior across developmental stages and held-out culture batches.

The extracted brightfield descriptors could provide an interpretable structural anchor for future multimodal organoid assessment. Brightfield imaging may support rapid, non-destructive monitoring during culture, whereas immunohistochemistry, multiplex immunofluorescence, mass spectrometry imaging, and other molecular measurements could provide higher-content endpoint characterization. Such integration could ultimately contribute to sensor-informed automated culture-monitoring workflows. However, no cross-modal association or multimodal prediction was evaluated here. Any added value would require appropriately paired or matched measurements and independent validation while accounting for developmental stage and culture batch [26,27,28].

### 4.7. Limitations and Implications

Several limitations should be considered. The six biologically independent culture batches represented distinct culture experiments, but all were generated in one laboratory and provided only six independent groups for evaluating cross-batch performance. Within-batch AUC was estimable for five all-stage and four maturation-only held-out batches: Batch 3 contained no maturation observations, whereas Batch 4 contained only QA-pass observations and therefore had no estimable AUC. These limitations in batch number and class composition, together with the wide conditional intervals and substantial variation among batches, do not establish robust generalization to other stem-cell lines, culture protocols, laboratories, or acquisition systems. Further evaluation using additional independent batches and external datasets is required.

The QA labels were assigned by one expert and retained the subjectivity inherent in routine visual assessment. Inter-rater agreement could not be assessed because independent QA ratings were unavailable. Future validation should include independent ratings from multiple experts to assess label reproducibility. Although initial ROI preparation was blinded, expert verification was not. Knowledge of the QA labels may have influenced ROI verification and consequently the extracted features and estimated classification performance. The images were two-dimensional brightfield projections acquired at low magnification and did not directly resolve cellular identity, molecular composition, electrophysiological function, or complete three-dimensional internal architecture. The repeated cross-sectional design also precluded inference about trajectories of individual organoids.

Only frozen ResNet-50 and DINOv2 encoders were evaluated as deep-feature baselines. The findings should therefore not be extended to task-specific fine-tuning, other pretrained encoders, or architectures developed specifically for organoid microscopy. Moreover, acquisition settings were not fully documented, and percentile normalization cannot exclude residual effects of exposure, gain, focus or illumination on the intensity-derived descriptors.

Within these constraints, the study provides a quantitative framework for translating routine brightfield QA observations into interpretable optical descriptors and for evaluating their dependence on developmental stage and culture batch. External validation across independent laboratories and acquisition settings, together with paired biological measurements, will be required before broader application.

## 5. Conclusions

This study shows that routine brightfield quality assessment of brain organoids can be translated into quantitative and interpretable optical descriptors. Across both the complete developmental dataset and the maturation-only analysis, the full handcrafted representation provided stronger discrimination than simple morphology and frozen ResNet-50 or DINOv2 features. The findings also demonstrate that developmental stage and culture batch must be considered explicitly, because QA failures were concentrated during maturation and performance varied across held-out batches. The QA-oriented ROIs were designed to retain visually relevant peripheral abnormalities, allowing leakage, debris, boundary disruption, internal heterogeneity, and component-level abnormalities to contribute directly to the analysis. It provides a quantitative structural baseline that may support future integration with spectroscopic, molecular, or other complementary sensing measurements.

This study presents an interpretable image-analysis approach for routine brightfield QA of brain organoids. Handcrafted features gave higher mean repeated-CV AUCs than the evaluated frozen DINOv2 and ResNet-50 pipelines, while held-out batch performance varied substantially. The primary handcrafted pooled LOBO AUC was 0.836, with a wide conditional interval, and sensitivity at the fixed 0.5 cutoff was 0.656. These findings support an exploratory baseline within the observed laboratory workflow. Validation with additional batches, independent QA ratings, blinded ROI verification and documented acquisition conditions is needed before broader use. Integration with spectroscopic or molecular measurements remains future work.

## Figures and Tables

**Table 1:** Composition of the revised all-stage and maturation-only analytical cohorts by culture batch. Counts refer to 692 image–ROI pairs after exclusion of two conflicting duplicate records. Zero maturation observations indicate that the subset was unavailable for that batch.

Culture Batch	All Stages			Maturation-Only		
	Total	QA-Pass	QA-Fail	Total	QA-Pass	QA-Fail
Batch 1	147	137	10	79	75	4
Batch 2	127	93	34	124	90	34
Batch 3	28	24	4	–	–	–
Batch 4	117	117	0	94	94	0
Batch 5	127	89	38	70	33	37
Batch 6	146	104	42	80	41	39
Total	692	564	128	447	333	114

**Table 2:** Repeated-CV values are mean seed-specific ROC AUC ± standard deviation across five seeds; these are descriptive summaries, not confidence intervals based on independent experiments. Pooled LOBO values show AUC and conditional 95% intervals obtained by resampling complete batches while holding fitted predictions fixed. LR: logistic regression; RF: random forest.

Scope	Representation	Classifier	Repeated CV	Pooled LOBO
			Mean AUC ± SD	AUC [95% Interval]
All stages	Handcrafted	LR	0.933±0.005	0.836 [0.625, 0.960]
All stages	Handcrafted	RF	0.926±0.005	0.797 [0.562, 0.918]
All stages	Frozen DINOv2	LR	0.892±0.011	0.782 [0.625, 0.863]
All stages	Frozen DINOv2	RF	0.885±0.004	0.715 [0.454, 0.832]
All stages	Frozen ResNet-50	LR	0.833±0.010	0.719 [0.627, 0.836]
All stages	Frozen ResNet-50	RF	0.856±0.005	0.629 [0.385, 0.789]
Maturation-only	Handcrafted	LR	0.895±0.005	0.778 [0.531, 0.940]
Maturation-only	Handcrafted	RF	0.888±0.006	0.767 [0.520, 0.901]
Maturation-only	Frozen DINOv2	LR	0.868±0.010	0.782 [0.694, 0.885]
Maturation-only	Frozen DINOv2	RF	0.868±0.007	0.612 [0.465, 0.745]
Maturation-only	Frozen ResNet-50	LR	0.815±0.011	0.627 [0.501, 0.748]
Maturation-only	Frozen ResNet-50	RF	0.822±0.008	0.461 [0.292, 0.677]

**Table 3:** Handcrafted logistic-regression operating-point performance under LOBO. QA-fail is the positive class. Metrics use the inherited score cutoff of 0.5, which was not optimized as a deployment threshold. TN, FP, FN and TP denote true negatives, false positives, false negatives and true positives. Results for the other representations and conditional intervals are provided in the supplementary metrics.

Scope	Sensitivity	Specificity	PPV	NPV	Balanced Accuracy	F1	FNR	FPR	TN/FP/FN/TP
All stages	0.656	0.869	0.532	0.918	0.763	0.587	0.344	0.131	490/74/44/84
Maturation-only	0.614	0.865	0.609	0.867	0.739	0.611	0.386	0.135	288/45/44/70

## Data Availability

The brightfield microscopy images and associated quality annotations supporting the findings of this study are available from the corresponding authors upon reasonable request. The Python and Bash scripts used for data preprocessing, feature extraction, model evaluation, and figure generation are publicly available at https://github.com/shunxingbao/interpretable-brightfield-organoid-qa (accessed on 15 September 2026).

## Abbreviations

The following abbreviations are used in this manuscript:

AI	Artificial intelligence
BH-FDR	Benjamini–Hochberg false-discovery rate
CI	Confidence interval
CV	Cross-validation
DINOv2	Self-distillation with no labels, version 2
GPU	Graphics processing unit
LOBO	Leave-one-batch-out
nnU-Net	Self-configuring U-Net-based biomedical image-segmentation framework
PCA	Principal component analysis
QA	Quality assessment
ResNet-50	50-layer residual network
ROI	Region of interest
ROC AUC	Area under the receiver operating characteristic curve
SAM	Segment Anything Model
ViT	Vision Transformer
